# Wistar rats choose alcohol over social interaction in a discrete-choice model

**DOI:** 10.1038/s41386-022-01526-8

**Published:** 2022-12-31

**Authors:** Gaëlle Augier, Veronika Schwabl, Asmae Lguensat, Mihai Atudorei, Osamudiamen Consoler Iyere, Sandra Eriksson Solander, Eric Augier

**Affiliations:** Center for Social and Affective Neuroscience, BKV, Augier lab, Linköping University, Linköping, 58185 Sweden

**Keywords:** Social neuroscience, Addiction, Reward

## Abstract

Animal models of substance use disorders have been criticized for their limited translation. One important factor behind seeking and taking that has so far been largely overlooked is the availability of alternative non-drug rewards. We recently reported that only about 15% of outbred Wistar rats will choose alcohol over a sweet solution of saccharin. It was also shown using a novel operant model of choice of drugs over social rewards that social interaction consistently attenuates self-administration and incubation of craving for stimulants and opioids. Whether this is also true for alcohol and choice of alcohol over a sweet reward translates to social rewards is currently unknown. We therefore evaluated choice between alcohol and a social reward in different experimental settings in both male and female Wistar rats. We found, in contrast to prior work that employed discrete choice of drugs vs. social reward, that rats almost exclusively prefer alcohol over social interaction, irrespective of the nature of the social partner (cagemate vs. novel rat), the length of interaction, housing conditions and sex. Alcohol choice was reduced when the response requirement for alcohol was increased. However, rats persisted in choosing alcohol, even when the effort required to obtain it was 10–16 times higher (for females and males respectively) than the one for the social reward. Altogether, these results indicate that the social choice model may not generalize to alcohol, pointing to the possibility that specific interactions between alcohol and social reward, not seen when a sweet solution is used as an alternative to the drug, may play a crucial role in alcohol vs. social choice experiments.

## Introduction

Animal models of substance use disorders (SUDs) have been criticized for their limited translation. Despite decades of research leading to significant progress in our understanding of the neurobiology of drug and alcohol addiction, advances in basic neuroscience have had limited impact on the treatment of addictive disorders. Promising mechanisms identified and validated in animal models of alcohol use disorder (AUD), such as the CRH1 receptor antagonism, failed in clinical trials [1–3]. This suggests that widely used animal models may not sufficiently account for the complexity of SUDs, leading on one hand to the though-provoking opinion that they may in fact have “impeded progress in treatments in humans” [4], but also on the other hand to the realization that preclinical models of addiction should be refined, rather than abandoned, to incorporate critical aspects of clinical addiction [5, 6].

One important factor behind seeking and taking that has so far been largely overlooked is the availability of alternative non-drug rewards [7, 8]. Addiction leads to a progressively increased choice of drugs over healthy rewards, such as social interactions [9–11]. Epidemiological studies have established a strong link between poor social integration and drug use [12]. Despite this, social factors and alternative to drugs have, with few exceptions, not been incorporated into neurobiological studies of addiction. To address this research gap, exclusive choice-based models between drugs and food reward have been operationalized. In both monkeys and rodents, operant responding for drug is reduced or prevented by the availability on an alternative non-drug reward, most of a time food pellets or a sweet solution [13, 14]. We have recently shown, in an investigation in which we screened over 600 rats for their choice between 20% alcohol and a sweet solution of saccharin, that this is also true for alcohol [15]. In parallel, Venniro and colleagues introduced a novel operant model of choice between drugs and social interaction [16] and reported that social interaction prevented methamphetamine self-administration and craving.

These rodent models of choice between drugs and social interaction have the potential to improve the translational validity and utility of existing preclinical models of addiction. However, it is currently unknown whether they also generalize to alcohol. Here, we therefore evaluated choice between alcohol and a social reward in different experimental settings. We first trained outbred Wistar male rats to “self-administer” 20% alcohol or the social reward (a social interaction with a cagemate) and assessed their preference when they were offered a mutually exclusive choice between alcohol and the social reward. We then investigated whether the nature of the partner rat, housing conditions (short or chronic isolation vs. group-housing) for both test and partner rats and sex could promote motivation for the social reward, as well as preference for social interaction over alcohol.

## Methods and materials

### Subjects

Adult male and female Wistar rats (Charles River, Germany, total *n* = 176, of which 48 were assigned as “social partner” (32 males and 16 females)), all weighing 180–250 g (8 weeks old of age) at the beginning of the experiments. Rats were grouped-housed (3–4 same sex rats per cage). In experiment 1, social partners and operant tested rats were housed in the same cage. Except for experiment 1 in which test subjects and partners were shifted (see below), social partners always remained operant- and drug-naïve and were housed separately from the test animals. The rats were maintained in a temperature- (21 °C) and humidity-controlled (45%) environment with a reversed 12 h light-dark cycle (off at 7:00 am, on at 7:00 pm). Rats were given free access to chow and tap water for the duration of the experiment. All behavioral testing was conducted during the dark phase of the light-dark cycle. Rats were weighed at least once a week. The studies were conducted in accordance with Swedish laws and approved by the Swedish Animal Ethics Committee (Jordbruksverket).

### Overview of experimental groups

A complete timeline of the experiments is provided in the corresponding figures.

#### Experiment 1: Effect of duration of the interaction and familiarity with partner

**Fig. 1:**
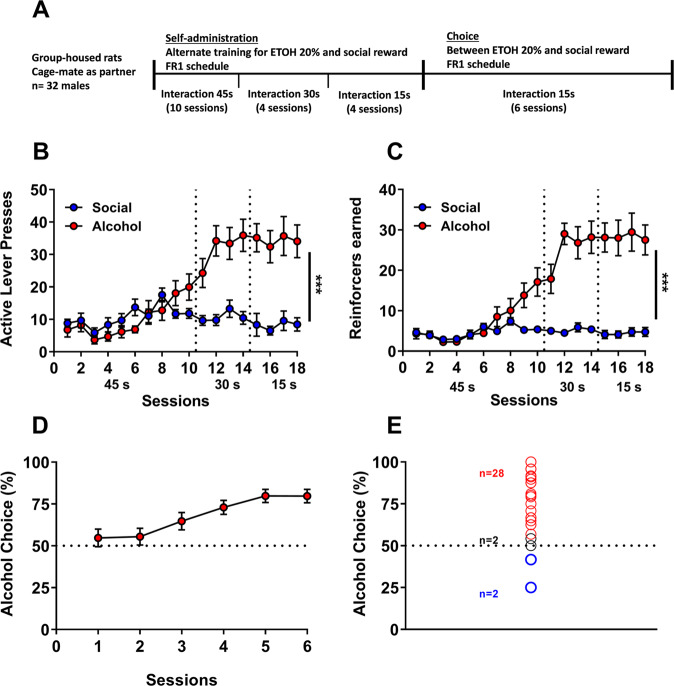
Rats prefer alcohol over social interaction with a cagemate, independently of the duration of the interaction. **A** Experimental Timeline for experiment 1 (**B**): Total number of active lever presses for the social reinforcer and alcohol across sessions. Tukey’s post-hoc comparisons: ***p < 0.001: significant differences compared with lever presses for social reward (**C**): Total number of reinforcers earned during sessions. Tukey’s post-hoc comparisons: ***p < 0.001: significant differences compared with the total number of earned social rewards (**D**): Percentage of alcohol choice across sessions using a discrete choice procedure. **E** Individual distribution of rats. **B**–**D** Data are expressed as mean ± SEM.

In this experiment, 32 male rats were used and group-housed (4 per cage: 2 test animals + 2 partner rats). Rats were first trained to self-administer either 20% (v/v) alcohol without sucrose/saccharin fading as described previously [17, 18] or to lever press for a social reward [19]. They were then offered a mutually exclusive choice between alcohol and the social reward, using a discrete-trials choice procedure [20] adapted from our previous choice procedure between alcohol and saccharin [15]. Detailed information is provided in Supplementary Methods section. At the end of this behavioral testing, partner and test subjects were switched in order to reduce the number of animals used. Since there was no difference between both groups for both training and choice, the results were pooled and shown together in Fig. 1.

#### Experiment 2: Effect of short and chronic isolation

A new cohort of rats (*n* = 32, group-housed) were used a test subject. An additional 16 rats (group-housed) were used as partner. Partner rats were from a different cohort and housed separately from the test rats. During the first part of the experiment, the test rats were divided in two subgroups: group-housed or short isolation 2 h before the experimental session (*n* = 16 each). During the second part of the experiment, the short isolation group became chronically isolated. We waited at least 2 weeks after chronic isolation to pursue behavioral testing.

#### Experiment 3: Does social interaction function as a reinforcer?

A new cohort of rats was used for this experiment. Both the test and partner rats were either group-housed or chronically isolated. Therefore, a total of 32 rats were used as test subjects in a 2 × 2 design (group-housed or chronically isolated with a group-housed or chronically isolated partner, *n* = 8 per group) and 16 rats as partners. Again, partner rats were from a different cohort and never housed with the test rats.

#### Experiment 4: Sex differences in motivation for social reward and social choice

A new cohort of 32 rats of both sexes (*n* = 16 per sex) were group-housed with individuals of the same sex. Partners were unknow rats (same sex), group-housed separately for the test rats.

## Results

### Rats prefer alcohol over social interaction with a cagemate, independently of the duration of the interaction

In an initial experiment, we trained rats on alternate days to lever press for alcohol or social interaction with a cagemate on a fixed-ratio 1 (FR1) reinforcement schedule. Once rats reached stable levels of responding, they produced more responses for alcohol (last three sessions: presses for alcohol: 34 ± 4.4; presses for social interaction: 8.2 ± 1.9) and earned more alcohol reinforcers compared to social reinforcers, regardless of the duration of social interaction (Fig 1). A two-way RM ANOVA of active lever presses data (Fig. 1B) indicated a main effect of the factors ‘sessions’ (*F*_(17,510)_ = 12.66, *p* < 0.001; eta^2^ = 0.30), ‘reinforcer: alcohol vs. social’ (*F*_(1,30)_ = 14.14, *p* < 0.001; eta^2^ = 0.32) as well as a significant interaction between these two factors (*F*_(17,510)_ = 13.57, *p* < 0.001; eta^2^ = 0.31). A post-hoc analysis showed that the total number of active presses for alcohol significantly exceeded presses for social rewards (*p* < 0.001) starting from session 12, and that decreasing the duration of the social interaction (from 45 to 15 s) did not affect pressing for the social reinforcer (*p* ≥ 0.89 for all sessions). The analysis of the number of reinforcers earned (Fig. 1C) supports the latter finding, given that both factors ‘sessions’ and ‘type of reinforcer’ were significant (*F*_(17,510)_ = 17.94, *p* < 0.001; eta^2^ = 0.37 and *F*_(1,30)_ = 42.63, *p* < 0.001; eta^2^ = 0.59, respectively) in addition to the interaction ‘sessions x type of reinforcer’ (*F*_(17,510)_ = 16.60, *p* < 0.001; eta^2^ = 0.36). Also, the number of earned alcohol rewards significantly surpasses the earned social rewards, starting from session 12 (Tukey HSD post-hoc*:* *p* < 0.001).

Rats were then offered daily sessions of mutually exclusive choice between alcohol and social interaction (Fig. 1D). Rats initially chose indifferently between the two rewards, but quickly started to shift toward alcohol (from 54.7 ± 5.2% of alcohol choice in session 1 to 79.7 ± 4.0% in session 6). At individual level (Fig. 1E), the vast majority of rats (28 out of 32: 87% of the population) preferred alcohol over social reward, while the few remaining rats were either indifferent (2 out of 32: 6%) or preferred social reward (2 out of 32: 6%).

### Rats choose alcohol over social interaction, even with a novel rat, regardless of housing conditions

#### The effects of a short isolation, 2 h prior to self-administration sessions

In a second experiment, we assessed whether housing conditions (i.e., short, or chronic isolation vs. group-housing) as well as maintaining the novelty of the social interaction (by using a novel rat instead of a cagemate as social partner) could promote motivation and choice for the social reward. A separate group of rats was trained on the alternating self-administration procedure under an FR1, followed by a FR2 schedule of reinforcement. Half the rats (*n* = 16) were isolated 2 h prior to the social self-administration session whereas the other half (*n* = 16) remained group housed. We found that a short isolation before the self-administration session did not affect pressing for the social and alcohol reward, or choice between the two reinforcers (Fig. S2).

#### The effects of a chronic isolation

**Fig. 2:**
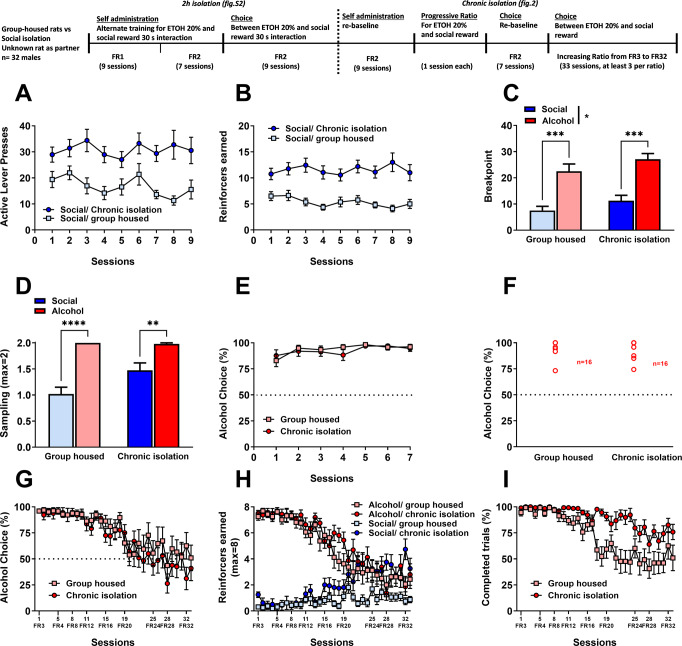
Rats choose alcohol over social interaction, even with a novel rat, regardless of housing conditions. **A** Total number of active lever presses for the social reinforcer in group housed and chronic isolation rats. **B** Total number of social reinforcers earned. **C** Mean breakpoint recorded during the progressive ratio sessions of reinforcement. Tukey’s post-hoc comparisons: ***p < 0.001 as significant differences compared with breakpoints for social reward. **D** Mean number of samplings displayed by group housed and chronically isolated rats for both alcohol and the social reinforcer. Tukey’s post-hoc comparisons: ***p < 0.001 and **p < 0.01 as significant differences compared with samplings for social reward. **E** Percentage of alcohol choice. **F** Individual distribution of grouped and chronically isolated rats. **G** Percentage of alcohol choice across sessions when increasing the fixed ratio. **H** Number of earned reinforcers (alcohol and social) across sessions when increasing the fixed ratio. **I** Percentage of completed trials across increased fix ratio requirement sessions. Data of (**A**–**E**) and (**G**–**I**) is expressed as mean ± SEM.

We then investigated whether chronic isolation would promote social choice (Fig. 2). To this end, rats were either chronically isolated, starting 2 weeks prior to additional behavioral testing, or remained group housed. We found that chronic isolation robustly potentiated the reinforcing properties of social interaction. Isolated rats responded more for the social interaction compared to group housed rats (Fig. 2A, last three sessions: 30.9 ± 4.3 vs. 13.5 ± 1.8 presses for social interaction respectively) and consecutively earned more social reinforcers (Fig. 2B). A two-way RM ANOVA of active presses for social interaction confirmed a main effect of the factor ‘housing condition’ (*F*_(1,30)_ = 14.18, *p* < 0.001; eta^2^ = 0.32) but only a trend for the factor ‘session’ (*F*_(8,240)_ = 1.81, *p* = 0.076) and no interaction between these two factors (*F*_(8,240)_ = 1.37, *p* = 0.21). Similarly, analysis of the reinforcers earned indicated a main effect of ‘housing condition’ (*F*_(1,30)_ = 23, *p* < 0.001; eta^2^ = 0.43) and a significant interaction ‘session x housing condition’ (*F*_(8,240)_ = 2.22, *p* < 0.05; eta^2^ = 0.07). Besides, post-hoc comparisons confirmed that chronically isolated rats obtained more social reinforcers during sessions 3, 4 and 6 to 9 (*p* < 0.05). We then assessed the motivation of the rats to obtain both rewards (social vs. alcohol), using a progressive-ratio schedule of reinforcement (Fig. 2C). Rats displayed a greater motivation for alcohol compared to social interaction, reflected by higher breakpoints (main effect of reinforcer type: *F*_(1,30)_ = 40.31, *p* < 0.001; eta^2^ = 0.57) and chronically isolated rats had a general increased motivation for both reinforcers (main effect of housing condition: *F*_(1,30)_ = 4.66, *p* < 0.05; eta^2^ = 0.13) compared to group housed rats. Post-hoc comparisons confirmed significantly higher breakpoints for alcohol compared to social reward (*p* < 0.001) for both group housed and chronically isolated rats.

In the discrete choice procedure, an in-depth analysis of the sampling phase confirmed the progressive ratio data. Rats performed more sampling trials for alcohol compared to social reward (main effect of reinforcer type: *F*_(1,30)_ = 57.14, *p* < 0.001; eta^2^ = 0.66) and chronically isolated rats generally earned more sampling for both rewards (main effect of housing condition: *F*_(1,30)_ = 5.30, *p* < 0.05; eta^2^ = 0.15, interaction: *F*_(1,30)_ = 5.83, *p* < 0.05; eta^2^ = 0.16) (Fig. 2D). Moreover, post-hoc analysis confirmed that sampling for alcohol was significantly higher in both chronically isolated (*p* < 0.01) and group housed rats (*p* < 0.001). However, despite its significant effect on social self-administration, motivation and sampling, chronic isolation did not promote choice of the social reward over alcohol (Fig. 2E) and both groups almost exclusively chose alcohol (last 3 sessions: 96.7 ± 1.7 and 96.2 ± 1.9% alcohol choice for the group housed and chronically isolated rats respectively). There was a main effect of session: *F*_(6,180)_ = 4.18, *p* < 0.001; eta^2^ = 0.12, but no effect of housing conditions (*F*_(1,30)_ = 0.12, *p* = 0.73) and no interaction between the two factors (*F*_(6,180)_ = 0.94, *p* = 0.47). Overall, all rats in both groups (group housed and chronic isolation) were alcohol preferent (Fig. 2F).

#### Rats persist in choosing alcohol, even when the cost is 14–16 higher than the one for social interaction

Finally, to quantify the relative magnitude of the alcohol reward and determine the point of indifference (i..e the ratio for which both the alcohol and social rewards are chosen equally), we used an increased fixed ratio schedule of reinforcement and progressively increased the ratio for alcohol between session, while the ratio for the social reward remained set at a FR2. We found that increasing the response requirement for alcohol significantly affected the percentage of alcohol choice in both group housed and chronically isolated rats (Fig. 2G, two-way RM ANOVA: main of session (*F*_(35,525)_ = 6.99, *p* < 0.001; eta^2^ = 0.32)). There was a trend for a main effect of housing conditions (*F*_(1,30)_ = 4.46, *p* = 0.051; eta^2^ = 0.23), as well as the significant interaction between session and housing conditions (*F*_(35,525)_ = 2.99, *p* < 0.001; eta^2^ = 0.17). Rats only started to reduce their alcohol choice on session 20, when the response requirement for alcohol was set to a FR20 (*p* < 0.01).

This effect was accompanied by a reduction of percentage of completed trials (Fig. 2I), in the group housed rats compared to chronically isolated rats, which maintained a relatively stable number of trials (two-way RM ANOVA: main effect of housing condition and sessions: *F*_(1,29)_ = 13.08, *p* < 0.01; eta^2^ = 0.31 and *F*_(35,1015)_ = 20.29, *p* < 0.001; eta^2^ = 0.42, respectively; as well as significant ‘housing condition × sessions’ interaction: *F*_(35,1015)_ = 5.41, *p* < 0.001; eta^2^ = 0.16). Also, Tukey post-hoc analysis of these data confirmed that group housed rats completed noticeably fewer trials starting from FR16 (session 17,18 corresponding to FR 16: *p* < 0.05 and *p* < 0.01; session 21,22 and 23 corresponding to FR 20: *p* < 0.05, *p* < 0.001 and *p* < 0.001, respectively).

An in-depth analysis of the absolute number of choice trials completed for each reward (Fig. 2H) confirmed that rats significantly decreased their number of alcohol choices throughout the sessions with increased ratio for alcohol (Three-way RM ANOVA: significant effect of ‘reinforcer type’, ‘housing condition’ and ‘sessions’: *F*_(1,59)_ = 68.33, *p* < 0.001; eta^2^ = 0.54; *F*_(1,59)_ = 5.06, *p* < 0.05; eta^2^ = 0.08 and *F*_(35,2065)_ = 9.93, *p* < 0.001; eta^2^ = 0.14, respectively). However, chronically isolated rats only started to shift their choice toward the social reward and reach indifference levels when the FR required to receive alcohol was increased to FR28. By contrast, the group housed rats continued to choose alcohol over social even at a FR32, although this resulted in less trials completed.

### Social interaction functions as a reinforcer in Wistar rats, dependently of the housing conditions of both test and partner rats

#### Social self-administration in alcohol-naïve rats

**Fig. 3:**
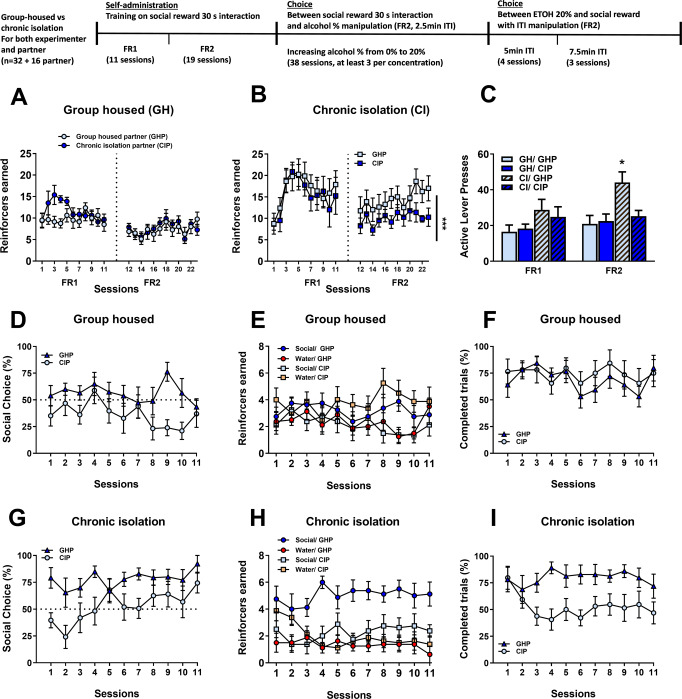
Social interaction functions as a reinforcer in chronically isolated rats. **A**, **B** Total number of social reinforcers earned in the group housed rats and chronic isolation rats respectively. **C** Total number of active lever presses for the social reinforcer in the group housed and chronic isolation rats. **D**–**G** Percentage of social choice vs. water in the group housed rats and chronic isolation rats respectively. **E**–**H** Number of earned reinforcers (water and social) across sessions earned in the group housed rats and chronic isolation rats respectively. **F**–**I** Percentage of completed trials across sessions in the group housed rats and chronic isolation rats respectively. Data of (**A**–**I**) is expressed as mean ± SEM.

In a third experiment, we investigated whether the alternate training procedure employed in experiments 1 and 2 may have devaluated social interaction as a reinforcer. We also assessed whether the housing conditions (chronic isolation vs. group-housing) of the partner rat could influence self-administration and choice for the social reward (Fig. 3). A separate group of alcohol- and operant-naïve rats was trained during 30 min sessions to lever press for social interaction under an FR1, followed by a FR2 schedule of reinforcement. Half the test rats (*n* = 16), as well as the partner rats (*n* = 8) were chronically isolated 2 weeks prior the initiation of operant training, whereas the other half (*n* = 16 and 8 respectively) remained group housed.

During the initial phase of training, we found in accordance with experiment 2 that chronically isolated rats self-administer more for social interaction than group-housed rats (Fig. 3A, B, *left panel*, last three sessions: 16.5 ± 2.5 and 15.4 ± 2.1 for chronically isolated rats with group housed (CI/GHP) and chronically isolated partner (CI/CIP) respectively vs. 9.7 ± 1.2 and 9.8 ± 1.2 for group housed rats with group housed (GH/GHP) and chronically isolated partner (GH/CIP) respectively) and showed a clear learning curve for social administration. A three-way RM ANOVA indicated a main effect of housing condition of the test rat: *F*_(1,29)_ = 10.86, *p* < 0.01; eta^2^ = 0.27). There was also a main effect of the factor ‘session’ (*F*_(10,290)_ = 5.46, *p* < 0.0001; eta^2^ = 0.16) and a significant interaction ‘session x housing condition’ (*F*_(10,290)_ = 3.29, *p* < 0.001; eta^2^ = 0.10). By contrast, the housing condition of the partner rat did not influence acquisition for social self-administration (no effect of housing condition, group-housed vs. chronically isolated: (*F*_(1,29)_ = 0.05, *p* = 0.83)). Once the ratio requirement was increased to FR2, we observed that only the chronically isolated rats paired with a group-housed partner (CI/GHP) increased their lever of responding (Fig. 3C) to maintain a high number of social reinforcers (Fig. 3A,B, *right panel*, last three sessions: 17.3 ± 2.7 for the CI/GHP group vs. 10.5 ± 1.3 for the CI/CIP group vs. 7.0 ± 1.0 and 8.0 ± 1.5 for the GH/GHP and GH/CIP groups respectively.

#### Choice between social interaction and water

To further test whether social interaction can act as a reinforcer in our experimental conditions, we performed daily sessions of mutually exclusive choice between water and social interaction (Fig. 3D–I). During the first session of choice, only the chronically isolated rats paired with a group-housed partner (CI/GHP) showed a clear preference for social interaction (79.2 ± 9.7% of social choice vs. 39.7 ± 7.0% for the CI/CIP vs. 56.5 ± 6.7% for the GH/GHP vs. 35.1 ± 9.6% for the GH/CIP). A three-way RM ANOVA confirmed that chronic isolation of the test rat promoted social choice (main effect of housing condition for test rats: *F*_(1,21)_ = 4.56, *p* < 0.05; eta^2^ = 0.18 as well as significant ‘housing condition for test rats × sessions’ interaction: *F*_(10,210)_ = 2.59, *p* < 0.01; eta^2^ = 0.11), whereas the opposite effect was seen for partner rats, in which group-housing instead of chronic isolation promoted social choice (main effect of housing condition of the partner: *F*_(1,21)_ = 10.29, *p* < 0.01; eta^2^ = 0.33 but no ‘housing condition of the partner × sessions’ interaction: *F*_(10,210)_ = 1.10, *p* = 0.36). Finally, there was no main effect of session (*F*_(10,210)_ = 1.01, *p* = 0.43), indicating that the rats quickly expressed their preference.

#### Choice between social interaction and increasing concentrations of alcohol

**Fig. 4:**
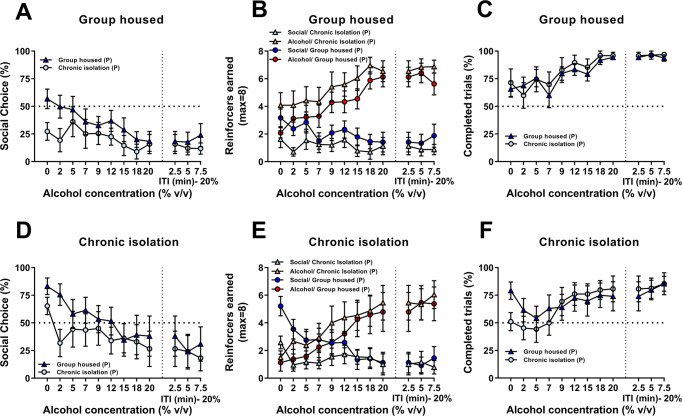
Effect of manipulating alcohol concentrations on social choice. **A**–**C** Percentage of social choice across sessions when increasing the % of alcohol and the length of the inter-trial in the group housed rats and chronic isolation rats respectively. **B**–**E** Number of earned reinforcers (alcohol and social) across sessions when increasing the % of alcohol and the length of the inter-trial in the group housed rats and chronic isolation rats respectively. **C**–**F** Percentage of completed trials across sessions when increasing the % of alcohol and the length of the inter-trial in the group housed rats and chronic isolation rats respectively. Data of (**A**–**F**) is expressed as mean ± SEM.

We then manipulated the magnitude of the drug reinforcer, by increasing the alcohol concentration during additional choice sessions to determine the concentration for which rats would choose social and alcohol equally, i.e., the point of indifference (Fig. 4A–F). As seen in the previous experiment, only the chronically isolated rats chose robustly social over water, while group housed rats were either indifferent (paired with a group housed partner) or prefer water (paired with a chronically isolated partner).

**Fig. 5:**
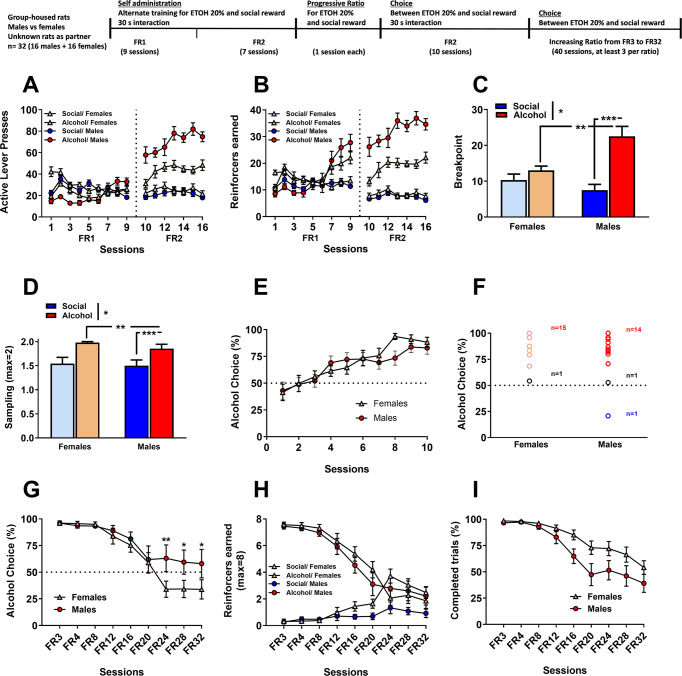
Sex differences in motivation for social reward and social choice. **A** Total number of active lever presses to get alcohol and the social reinforcer of male and female rats. **B** Total number of reinforcers earned. **C** Mean breakpoint recorded during the progressive ratio sessions of reinforcement. Tukey post-hoc comparisons: ***p < 0.001 as significant difference compared with breakpoints for social reward for males. **p < 0.001 as significant difference compared with breakpoints for alcohol expressed by for females. **D** Mean number of samplings displayed by male and female rats for both alcohol and the social reinforcer. Tukey post-hoc comparisons: ***p < 0.001 as significant difference compared with samplings for social reward for males. **p < 0.001 as significant difference compared with samplings for alcohol expressed by for females. **E** Percentage of alcohol choice. **F** Individual distribution of male and female rats. **G** Percentage of alcohol choice across sessions when increasing the fixed ratio. Tukey post-hoc comparisons: **p < 0.01 and *p < 0.05 as significant difference compared with the percentage of choice showed by females. **H** Total number of earned reinforcers (alcohol and social) across sessions when increasing the fixed ratio. **I** Percentage of completed trials across increased fixed ratio requirement sessions. Data of (**A**–**E**) and (**G**–**I**) is expressed as mean ± SEM.

At 2% alcohol already, all group started to drop their social choice (75.5 ± 10.9% of social choice for the CI/GHP vs. 32.4 ± 15.2% for the CI/CIP vs. 49.7 ± 11.6% for the GH/GHP vs. 16.2 ± 9.6% for the GH/CIP) and only the CI/GHP rats kept choosing social over a low concentration of alcohol. A three-way RM ANOVA analysis confirmed a main effect of alcohol concentration (*F*_(8,232)_ = 12.60, *p* < 0.0001; eta^2^ = 0.30), a main effect of housing condition for test rats (*F*_(1,29)_ = 2.71, *p* < 0.05; eta^2^ = 0.10) but no main effect of the factor ‘housing condition of the partner rats (*F*_(1,29)_ = 1.65, *p* = 0.21) or any significant interaction between the factors (*p* ≥ 0.14 for all interactions). The latter result indicates that manipulating the alcohol concentration mostly affected all groups similarly. In addition, a Tukey post-hoc analysis showed that overall, social choice was significantly different when reaching an alcohol concentration of 15% (*p* < 0.01 for the 15%, 18% and 20% alcohol concentrations), an effect that is mostly driven by the CI/GHP group that for the first time showed a clear preference for alcohol at this concentration (28.7 ± % of social choice for the CI/GHP vs. 36.1 ± 14.1% for the CI/CIP vs. 28.8 ± 11.6% for the GH/GHP vs. 14.5 ± 9.3% for the GH/CIP). Finally, increasing the alcohol concentration significantly affected the percentage of completed trials during choice for all groups (main effect of alcohol concentration (Fig. 5C and F: *F*_(8,232)_ = 12.87, *p* < 0.0001; eta^2^ = 0.31 but no effect of other factors or interactions), with rats completing more trials when they were offered a choice between social interaction and high concentrations of alcohol (*p* < 0.05 for the 12% and higher alcohol concentrations).

Of note, we found that increasing the ITI did not affect preference for alcohol (no main effect of the “ITI”, “housing conditions for test rats” and “housing conditions for partners” factors; *p* ≥ 0.21 for all factors).

### Sex differences in motivation for social reward and social choice

Finally, in this last experiment, we examined potential sex differences in motivation and choice for the social reward. To this end, we trained a different group of rats (*n* = 16 males, *n* = 16 females) on an alternated self-administration procedure. We found no difference in self-administration for the social reward. However, males earned almost twice more alcohol reinforcers compared to females (last 3 sessions: 35.1 ± 1.9 and 20.5 ± 1.3; Fig. 5A, B: *see supplementary text* for full analysis).

Analysis of the breakpoints reached during the PR sessions (Fig. 5C) indicated a main effect of the reinforcer type (social vs. alcohol, two-way ANOVA: *F*_(1,30)_ = 18.87, *p* < 0.001; eta^2^ = 0.39), a trend for a general effect of sex (*F*_(1,30)_ = 3.48, *p* = 0.072; eta^2^ = 0.10) and a significant interaction between both factors (*F*_(1,30)_ = 9.14, *p* < 0.01; eta^2^ = 0.23). In addition, Tukey post-hoc analysis showed that males were more motivated (higher breakpoints) to obtain alcohol compared to social rewards (*p* < 0.001) and compared to females (*p* < 0.01).

In the discrete choice procedure, rats from both sexes completed more sampling trials for alcohol compared to social reward (Fig. 5D), with a main effect of the factor reinforcer type (two-way ANOVA, *F*_(1,30)_ = 26.85, *p* < 0.001; eta^2^ = 0.47). Tukey post-hoc analysis showed that both males and females significantly sampled more alcohol than the social reward (*p* < 0.001).

In the choice trials, both males and females almost exclusively chose alcohol over social interaction at a comparable rate (Fig. 5E: no main effect of sex (*F*_(1,30)_ = 0.21, *p* = 0.65)) and showed a similar group distribution, with the majority of rats being alcohol preferring (females: 15 out of 16: 93%, males: 14 out of 16: 87%) (Fig. 5F).

Moreover, while we observed that increasing the FR required to get alcohol significantly affected alcohol choice rates in both sexes, this was not sufficient to shift preference toward social interaction in males (males: from 96.1 ± 2.0% of alcohol choice at FR3 to 58.1 ± 13.3% at FR32, females: from 96.0 ± 2.2% of alcohol choice at FR3 to 33.9 ± 9.0% at FR32) (Fig. 5G). A two-way RM ANOVA indicated a main effect of both factors ‘sex’ and ‘FR’ (*F*_(1,30)_ = 7.80, *p* < 0.05; eta^2^ = 0.28 and *F*_(8,240)_ = 17.32, *p* < 0.001; eta^2^ = 0.46, respectively). In addition, Tukey post-hoc analysis showed significant differences between the percentage of preference expressed by males and females at FR24 (*p* < 0.01), FR28 (*p* < 0.05) and FR32 (*p* < 0.05). Analysis of reinforcers earned indicated that females shifted toward choosing more social rewards at FR24 (Fig. 5H), but the number of completed trials showed a trend for males to complete fewer trials than females ((Fig. 5I; two-way RM ANOVA: sex factor: *F*_(1,30)_ = 4.02, *p* = 0.053; eta^2^ = 0.12). Moreover, the increase of FR required to receive alcohol significantly reduced the number of completed trials (FR factor: *F*_(8,240)_ = 39.92, *p* < 0.001; eta^2^ = 0.57 and interaction ‘FR x sex’: *F*_(8,240)_ = 2.33, *p* < 0.05; eta^2^ = 0.07).

## Discussion

We used an operant procedure in which rats are allowed a mutually exclusive choice between alcohol and an alternative non-drug reward, a social interaction with a partner rat. Unexpectedly, we found that outbred Wistar rats almost exclusively responded for alcohol when offered the opportunity to access the social reward as an alternative. This preference for alcohol over social interaction was maintained, independently of the nature of the social partner (cagemate vs. novel rat), the length of social interaction, housing conditions (group housed vs. short isolation before the operant session or chronic isolation) or sex.

### Rats choose alcohol over social interaction, by contrast with previous choice literature

Our findings are in contrast to prior work that employed discrete choice of drugs vs. social reward, in which the availability of social interaction reduced self-administration of methamphetamine [16], heroin [21], remifentanil [22] and cocaine [23, 24]. In addition, social choice-induced abstinence also prevented the incubation of craving typically observed after forced-abstinence from these drugs. However, in a recent investigation independently performed at the same time, Marchant et al. also found that rats will choose alcohol over social interaction [25].

Why these results do not generalize to alcohol remains unclear at this point. A major difference between our study and previous literature on social choice is the route of administration of the drug reward. Indeed, similar to the vast majority of preclinical alcohol research, rats in our study self-administered alcohol orally, whereas the opioids and stimulants used to establish the social choice model have been self-administered intravenously. We believe, however, that this is unlikely to account for the contradictory findings, since our main finding that rats will strongly prefer alcohol over social interaction, independently of several experimental settings, is also in contradiction with our previous work that has investigated the molecular substrates of choosing alcohol over a sweet solution of saccharin [15]. Using a discrete, mutually-exclusive choice procedure similar to the one used in the present investigation, we characterized over 600 Wistar rats for their choice behavior and found that, in accordance with previous work with other drugs of abuse that are self-administered intravenously [26–29], most of them quickly stop responding for alcohol when they are given access to a potent alternative reward, a sweet solution of saccharin. In that study, we also found that about 15% of outbred rats persist in choosing alcohol over an alternative high-value reward, a rate similar to human alcohol addiction [30]. In contrast, when evaluating choice preferences using the social choice model, 92.5% of males and 94% of females robustly favored the alcohol reward, and half of the remaining rats were indifferent.

Another methodological difference that could explain our surprising findings is that we used Wistar rats in the present study whereas Sprague-Dawley rats were used in most previous social choice work. Although we found that social interaction clearly functions as an operant reinforcer in Wistar rats, as shown by a clear learning curve for social self-administration, increased lever responding when the FR requirement is increased to FR2 and a strong preference for social over water or very low alcohol concentration (at least in chronically isolated rats paired with a group-housed partner), it will be important to test the generality of our alcohol/social choice findings with other rat strains, in particular Sprague-Dawley, in a subsequent study. Of note, Long Evans rats will also choose alcohol over social interaction, irrespective of sex and manipulations of the alcohol reinforcer [25]. Finally, this apparent discrepancy could also simply reflect the fact that social reward may act as a weaker reinforcer than the sweet solution of saccharin that we employed in previous studies. In agreement, it was recently shown that rats strongly prefer palatable food over social interaction [22].

### Interactions between alcohol and social reward

Collectively, these observations point to the possibility that specific interactions between alcohol and social reward, not seen when a sweet solution is used as an alternative to the drug, may play a crucial role in alcohol vs. social choice experiments. Previous literature suggests that the interaction between AUD and social factors are quite complex with both negative and positive effects on alcohol use and relapse. For example, studies investigating the effect of proximal social factors on alcohol self-administration reported that the presence of a social partner promotes alcohol operant self-administration and drinking in different experimental settings (isolation and group housing, social subordination, presence of an intoxicated partner) [31–33]. This suggests that, as opposed to psychostimulants [16, 23, 34] and opioids [21, 22], alcohol self-administration and choice could promote each other in a social context.

A potential confounding factor to our initial observation that rats will choose alcohol over social interaction is that, in our first experiments, social interaction appears to be a weaker reinforcer than in previous literature. This could be explained by several factors, including methodological differences. Here, for example, we performed 30 min long session of social self-administration (to keep the same length for both alcohol and social rewards), compared to 1- or 2 h long sessions in the recent work by Chow et al. for example [22]. Contrary to alcohol, for which a front-loading phenomenon is typically observed and most of the reinforcers are typically obtained during the first 15 mins of the sessions [35], social self-administration remains equally spaced during the session. Therefore, it would be expected that rats would earned almost a double number of reinforcers if we trained them during 1 h sessions instead.

It is also possible that our alternate training procedure with both alcohol and social may have devaluated social interaction. To test this hypothesis, we trained an independent group of rats (Fig. 3) to self-administer social only, with no prior experience to alcohol or operant behavior. We found that social interaction clearly functions as an operant reinforcer in Wistar rats, as shown by a clear learning curve for social self-administration, increased lever responding when the FR requirement is increased to FR2 and strong preference for social over water or very low alcohol concentration. At least when rats are kept within the appropriate housing conditions, i.e., isolated rats with group partners. These rats obtained 17.3 social reinforcers on a FR2 during 30 min sessions, a number that is similar to the reinforcers earned by rats with 1 h access to social self-administration in a recent study [22]. Despite this, rats only choose social over alcohol at very low alcohol concentrations.

### The effect of housing conditions and sex on social reward and choice of alcohol over social

Our study also highlights that chronic isolation promotes the motivation for social interaction, since isolated rats self-administered more and reached higher breakpoints for the social reward compared to group housed rats. This result aligns with previous research reporting that isolation promotes social seeking and interaction [22, 36] and that neuronal activation of the Nucleus Accumbens shell is associated with social seeking after social isolation [37].

Nevertheless, this was insufficient to influence alcohol choice, and both group housed and chronically isolated rats displayed high and almost exclusive alcohol choice. They persisted in choosing alcohol, even when the effort required to obtain it was 14–16 times higher than the one for the social reward. This could be explained by the fact that, in addition to its effect on social interaction, social isolation can also act as a stressor, promoting drug self-administration and alcohol-related behaviors [38–41]. Moreover, it has been reported that the presence of a partner rat during drug self-administration acts as a reinforcer, when presented as a consequence of pressing for the drug, promoting thus drug intake [42]. In our experimental settings, however, choosing alcohol meant that the opportunity for social interaction was dismissed at the expense of the drug, potentially suggesting a lower reinforcing value of social interaction when presented with alcohol.

Finally, we observed that males rats robustly self-administer more alcohol and exerted a higher motivation for alcohol compared to female rats, in agreement with a recent report indicating that males subjected to intermittent access to alcohol display higher alcohol intake levels than females [43]. In contrast, we found no sex differences in operant responding and motivation for the social reward. During the choice procedure itself, we only found subtle sex differences, with overall, both males and females robustly choosing alcohol over social reward. However, females shifted toward the social reward earlier when the response requirement was increased for alcohol (FR20), and completed more trials.

### Concluding remarks

Altogether, our results, as well as the recent findings from Marchant et al. [25], indicate that the social choice model may not generalize to alcohol, pointing to the possibility that specific interactions between alcohol and social reward, not seen when a sweet solution is used as an alternative to the drug, may play a crucial role in alcohol vs. social choice experiments. This surprising outcome appears to be robust as evidenced by its reproducibility in two separate labs with two different strains of rats. These results also highlight the importance of taking in account the housing conditions of both test and partner rats in social choice studies.

## Supplementary information

Supplementary method and figures
