# Supplementary material for: Wistar rats choose alcohol over social interaction in a discrete-choice model
Source: Neuropsychopharmacology. 2022 Dec 31;48(7):1098–107. doi: 10.1038/s41386-022-01526-8 (PMC10209174; doi:10.1038/s41386-022-01526-8)
Supplement: Supplementary file 1 — Supplementary method and figures [file 41386_2022_1526_MOESM1_ESM.docx]

Supplementary Materials for

**Wistar rats choose alcohol over social interaction in a discrete-choice model**

Gaëlle Augier^1^, Veronika Schwabl^1^, Asmae Lguensat^1^, Mihai Atudorei^1^, Osamudiamen Consoler Iyere^1^, Sandra Eriksson Solander^1^, Eric Augier^1+^

**Affiliations:**

^1^ Center for Social and Affective Neuroscience, BKV, Augier lab, Linköping University, Linköping, 58185, Sweden

+ Correspondence to: Eric Augier (eric.augier@liu.se)

**Supplementary Methods and Materials**

**Behavioral equipment**

Operant training and testing was performed in sixteen identical operant chambers (Med Associates Inc., St Albans, VT, USA; 30.5×29.2×24.1 cm), custom-made to include a social-partner chamber, as described elsewhere [1]. Each operant chamber was equipped with a compartment for a social peer, connected to the chamber through an automatic sliding door (Supplementary Fig. 1). Two retractable levers were positioned laterally to a dual liquid cup receptacle, and a third one was positioned next to the door. A metal grid barrier was placed in front of the door to avoid the partner rat from escaping and entering the operant chamber during the ongoing sessions. In most experiments, rats underwent an alternate training for both the alcohol and social rewards before being offered a mutually exclusive choice between alcohol and social interaction.

**Alcohol self-administration**

Operant- and drug-naive rats were trained to self-administer 20% (v/v) alcohol without sucrose/saccharin fading as described previously [2, 3]. Briefly, rats were first trained on a fixed-ratio (FR) 1 5s time-out (TO) schedule to self-administer 20% alcohol during 30 minute sessions. At the onset of the 30-min length session, a red cue-light located above the lever associated with alcohol reward was illuminated for 30s to signal the beginning the alcohol session, after which the lever was extended. Pressing once was reinforced by the delivery of a volume of 100 microliters of 20% alcohol in water in the adjacent drinking well and initiated a concomitant 5 second time-out period signaled by the illumination of the cue-light above the lever. Responses during the time-out period were recorded but had no programmed consequences. Sessions were conducted 5 days a week until stabilization of performance (no change greater than 15 % in the total number of reinforcers earned during the last 3 sessions). Except for experiment 1, once a stable self-administration baseline was reached, the ratio was increased to an FR2 and more sessions were performed until stabilization of performance.

**Social self-administration**

On alternate days to alcohol self-administration, rats were trained to lever press for a social reward. Before entering the chamber, the partner rat was placed in the social box adjacent to the operant chamber. Except for experiment 1 (in which the partner rat was a cagemate), partner rats were always housed in a different homecage than the operant trained rats, and a new partner was presented for each session to maintain the novelty of social interaction (since we had 16 partners, the same rat was not presented before next 16 session). At the onset of the 30-min length session, a green cue-light located above the lever associated with social reward was illuminated for 30s to signal the beginning of the social session, after which the lever was extended. Pressing once was reinforced by the opening of the automatic sliding door for a limited time and the illumination of the associated cue-light. For experiment 1, we tested three different length of social interaction (45s, 30s or 15s). For all other experiments, we set this time of interaction at 30s. At the end of the social interaction time, the guillotine door automatically closed, and this was followed by a 5 second time-out period during which responses on the lever were recorded but were without programmed consequences, paralleling the alcohol self-administration protocol. Once a stable self-administration baseline was reached (no change greater than 15 % in the total number of reinforcers earned during the last 3 sessions), the fixed ratio was increased and additional FR2 sessions were performed until stabilization of performance.

**Discrete-choice procedure**

Trained rats were then offered a mutually exclusive choice between alcohol and the social reward, using a discrete-trials choice procedure [4] adapted from our previous choice procedure between alcohol and saccharin [5]. At the onset of the choice session, both the red cue-light previously associated with alcohol reward and the green cue-light previously associated with social reward were illuminated for 30s to signal the beginning of choice session. A choice session consisted of a total of 12 trials, spaced by a 2.5 minute inter-trial interval (ITI) and was divided into two distinct phases. First, during a sampling phase, a trial started by the presentation of one single lever (the alcohol- or social-paired lever) and rats were therefore allowed to sample each reinforcer separately on a FR1 or FR2 depending on the experiment (twice for alcohol and twice for social, amounting to a total of 4 trials). Responding within 2.5 minutes on the lever presented led to the retraction of that lever and was reinforced by the delivery of a volume of 100 microliters of 20% alcohol in water in the adjacent drinking well, or by the opening of the guillotine door allowing a 30s social interaction (only 15s for the experiment 1) and initiated a concomitant 5 second time-out period signaled by the illumination of the cue-light above the lever. If rats failed to respond within 2.5 minutes, the lever was retracted, and no reinforcer was delivered.

The sampling phase was followed by a mutually exclusive choice phase, during which both reinforcers were presented at the same time. Each trial started by the simultaneous presentation of both alcohol- and social-paired levers, and rats could choose between the reinforcers. Responding within 2.5 minutes on one of the levers led to the associated reinforcer being delivered, and retraction of both levers until the next trial was initiated. Accessing the other reinforcer was therefore not possible until the next trial. If rats failed to respond within 2.5 minutes, both levers were retracted, and no reinforcer was delivered. To avoid accidental lever presses to be reinforced as choices, the response requirement was set to two consecutive responses. A response on the alternate lever before completion of the response requirement reset it. Daily choice sessions were performed until a stable preference was observed (no change greater than 15 % in the total number of reinforcers earned during the last 3 sessions). The preference of the animals was expressed as the percentage of the total number of alcohol choice over the total number of choices (alcohol + social).

**Increased Fixed Ratio choice procedure**

In contrast to the discrete-choice procedure, where rats had to press a similar amount of time on both levers associated with the alcohol and social rewards, we gradually increased the ratio (3, 4, 8, 12, 16, 20, 24, 28, 32) for the preferred reward (i.e., alcohol), whereas the response requirement for social interaction was maintained to an FR2. The ratio for alcohol was increased only after stabilization of response was obtained (at least 4 sessions per ratio).

**Progressive ratio schedule of reinforcement**

The motivation of the animals to consume alcohol and to press for social reward and alcohol was assessed using a progressive ratio schedule [6]. Conditions were identical to baseline self-administration except that the response requirement to receive the reinforcer was increased within-session according to the following formula: 1, 2, 3, 4, 6, 8, 10, 12, 16, 20, 24, 28, 32…. The self-administration session terminated once 30 minutes had elapsed without a reinforcer. The breakpoint was defined as the last completed response requirement during the progressive ratio test. The order of the progressive ratio sessions was counterbalanced between the groups (i.e. half of the animals were tested for alcohol on day 1, the other half for social and vice versa on day 2).

**Statistical analysis**

Data were first examined for significant violations to the assumption of homogeneity of variances using Levene’s test. Since no deviation from homogeneity of variances was detected, all data were analyzed using analysis of variance (ANOVA), with factors for the respective analysis indicated in conjunction with its results. Choice data are presented in percentage to facilitate the readability of the figures. All *post-hoc* analyses were conducted when appropriate using a Tukey-HSD test. The accepted level of significance for all tests was p <0.05.

**Supplementary Fig. 1 Operant social box.**


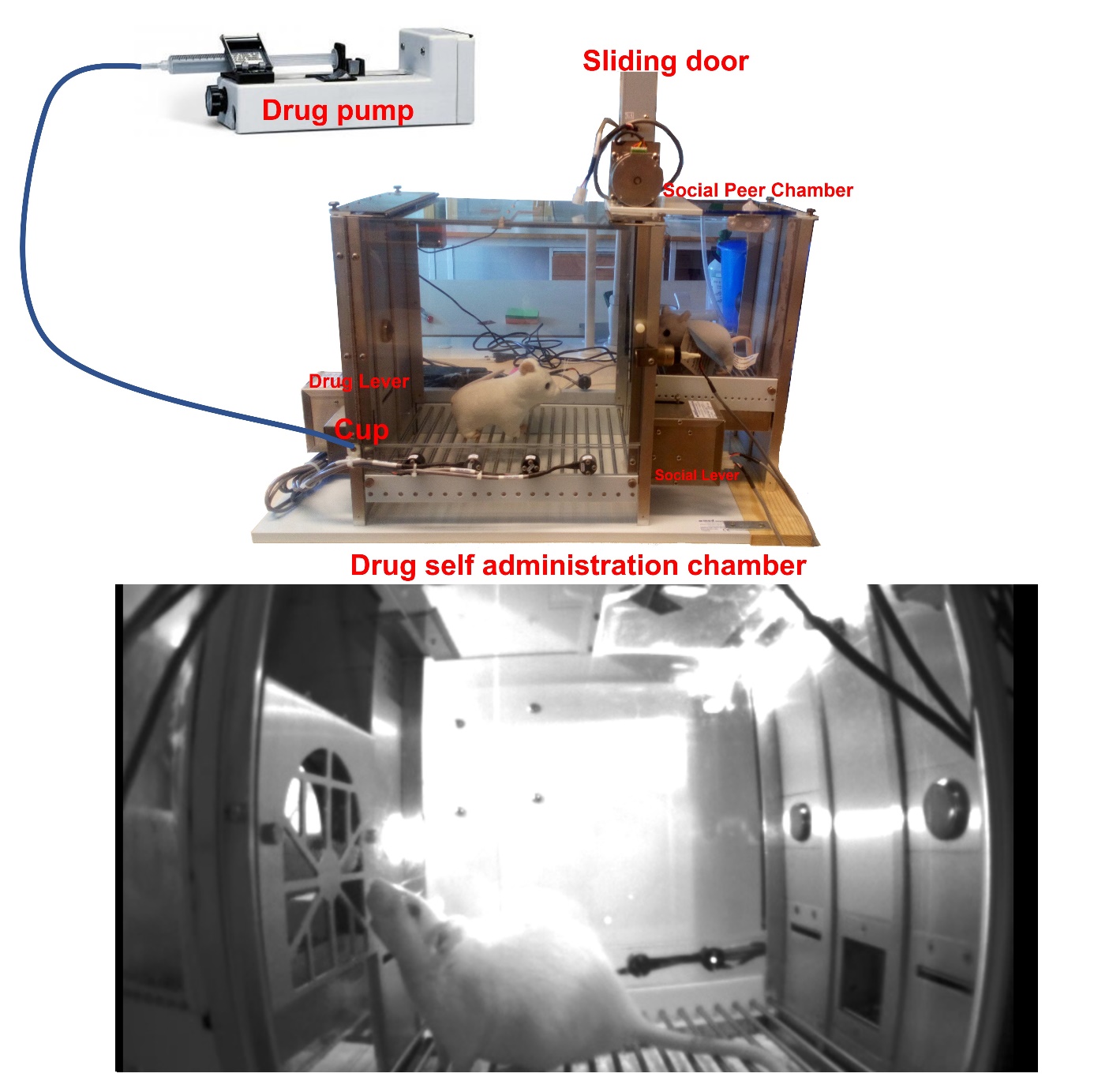


**Supplementary Fig. 2 Rats choose alcohol over social interaction, despite a short isolation before the operant session**

**(A)**: Total number of active lever presses for the social reinforcer and alcohol across FR1 and FR2 sessions. Tukey’s *post-hoc* comparisons: ***: p< 0.001: significant differences compared with lever presses for social rewards **(B)**: Total number of reinforcers earned during FR1 and FR2 sessions. Tukey’s *post-hoc* comparisons: ***: p< 0.001: significant differences compared with the total number of earned social rewards **(C)**: Percentage of alcohol choice of group housed and 2h isolated rats across the discrete choice procedure sessions. **(D)**: Individual distribution of grouped and 2h isolated rats. Panels **A-C** data is expressed as mean ± SEM.


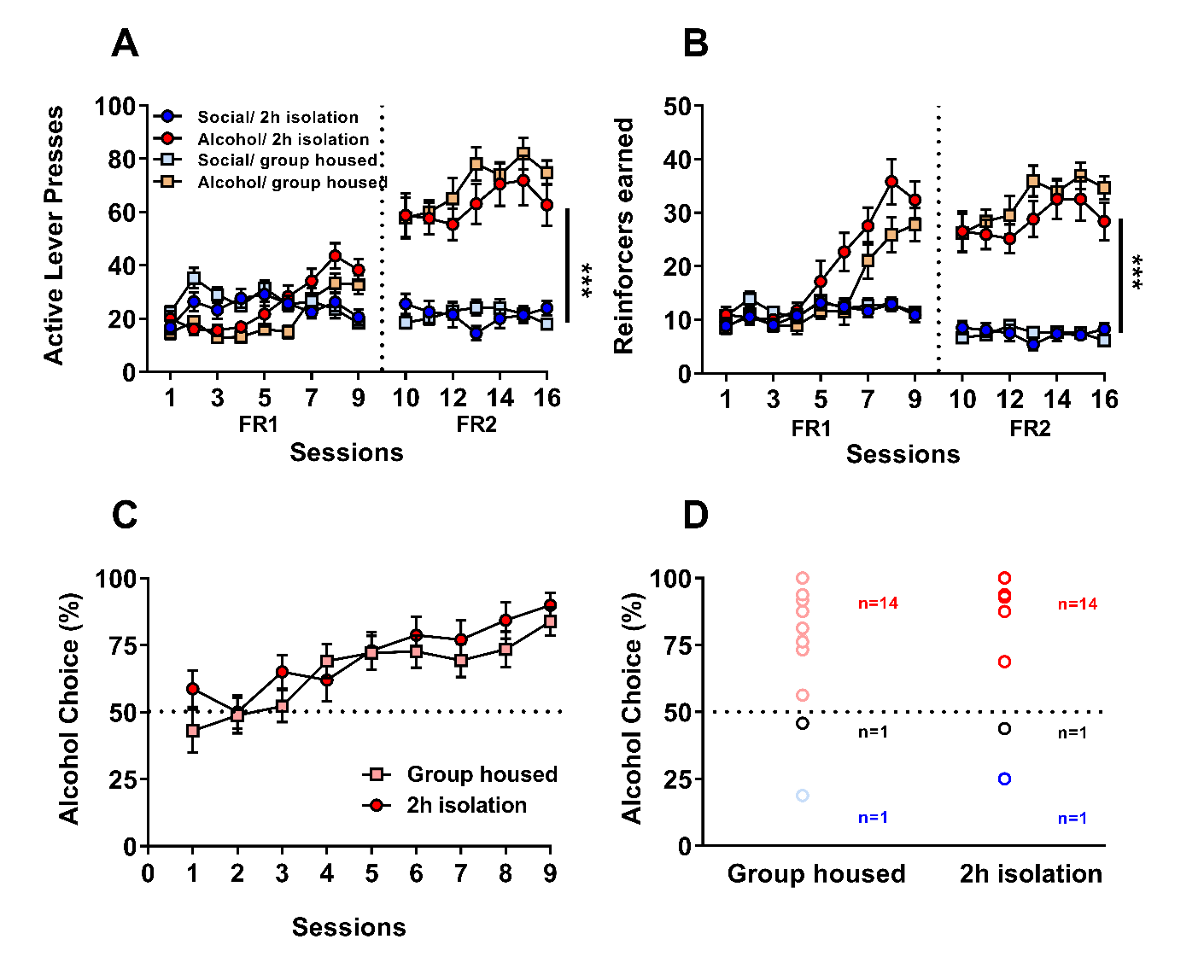


Similarly, to experiment 1, rats pressed more for alcohol and choose alcohol over social interaction, regardless of their housing conditions (group housed *vs.* 2h isolation) **(Supplementary Fig2)**. During acquisition (FR1 schedule of reinforcement, **Sup** **Fig 2A**), rats quickly initiated responses at similar rates for both alcohol and social interaction (main effect of the factor ‘session’ (F _(8,472)_ =9.71, p<0.001; eta^2^=0.14), but no significant effect of ‘reinforcer type’ and ‘housing condition’ (F _(1,59)_ =0.56, p=0.46 and F _(1,59)_ =0.93, p=0.33, respectively)). Moreover, the interactions ‘session x reinforcer type’ and ‘session x housing condition’ were significant (F _(8,472)_ =20.56, p<0.001; eta^2^=0.26 and F _(8,472)_ =2.63, p<0.01; eta^2^=0.04, respectively). Concerning FR2 sessions data, the same analysis indicated a main effect of both factors ‘session’ (F _(6,366)_ =3.32, p<0.01; eta^2^=0.05) and ‘reinforcer type’ (F _(1,61)_ =131.83, p<0.001; eta^2^=0.68). The remaining factor was not statistically significant (‘housing condition’: F _(1,61)_ =0.87, p=0.36). In contrast to FR1 data, only the interaction ‘session x reinforcer type’ was significant (F _(6,366)_ =3.90, p<0.001; eta^2^=0.06). Moreover, Tukey’s *post-hoc* multiple comparisons pointed out significantly higher total active presses for alcohol compared to social reward, during all FR2 sessions (p<0.001).

Rats also earned significantly more alcohol reinforcers during both FR1 and FR2 sessions **(Sup Fig 2B).** A three-way RM ANOVA analysis of FR1 data, with ‘session’, reinforcer type’ and ‘housing condition’ as factors showed a main effect of the factors ‘session’ and ‘reinforcer type’ (F _(8,472)_ =28.54, p<0.001; eta^2^=0.33 and F _(1,59)_ =21.65, p<0.001; eta^2^=0.27, respectively). Besides, the interactions ‘session x reinforcer type’ and ‘session x housing condition’ were also significant (F _(8,472)_ =22.59, p<0.001; eta^2^=0.28 and F _(8,472)_ =2,30, p<0.05; eta^2^=0.04, respectively)­. Similarly, FR2 data analysis showed a main effect of session (F _(6,366)_ =3.25, p<0.01; eta^2^=0.05) and reinforcer type (F _(1,61)_ =182,44, p<0.001; eta^2^=0.75), in addition to the interaction between both factors (F _(6,336)_ =4.58, p<0.001; eta^2^=0.07). However, the housing condition had no effect (F _(1,61)_ =1.11, p=0.30). Besides, the total number of earned alcohol rewards significantly surpasses the number of social rewards (Tukey’s *post-hoc* comparisons: p<0.001). During the first session of the discrete choice procedure **(Sup Fig 2C)**, rats (group housed and 2h isolation) chose similarly between alcohol and social interaction. However, they quickly diverge from the indifference threshold (50%) and reach 83.8± 5.12 and 89.8±4.56 % alcohol choice by session 9, respectively. Overall, both the group housed and the 2h isolated rats displayed similar alcohol choice rates (Two-way RM ANOVA: main effect of sessions: F _(8,240)_ =17.70, p<0.001; eta^2^=0.37, but no effect of housing condition: F _(1,30)_ =0.67, p=0.42).Moreover, the individual distribution between the two groups was similar **(Sup Fig 2D)**, given that 14 out of 16 rats in each group (87%) were alcohol preferent, 1 out of 16 (6%) chose indifferently between alcohol and social interaction and only 1 out of 16 (6%) favored the social reward. All together, these results indicate that a short isolation before the self-administration session is not sufficient to promote the reinforcing properties of the social interaction.

**Supplementary Text for Fig. 5**

We found no difference in the total number of active lever presses between males and females during the FR1 acquisition, and both sexes initiated operant self-administration at a similar rate **(Fig 5A, left)**. In more details, for the social reward, two-way RM ANOVA indicated a main effect of sessions (F _(8,232)_ =8.36, p<0.001; eta^2^=0.22) but no main effect of sex (F _(1,29)_ =2.61, p=0.12). Similarly, for the total number of presses for alcohol, there was a main effect of sessions (F _(8,240)_ =9.99, p<0.001; eta^2^=0.25), a significant interaction between sessions and sex (F _(8,240)_ =4.68, p<0.001; eta^2^=0.14) but no main effect of sex (F _(1,30)_ =1.25, p=0.27). Once the response requirement was increased to FR2 **(Fig 5A, right)**, the same analysis confirmed that males and females pressed to a similar extent for the social reward (no main effect of sessions (F _(6,180)_ =1.51, p=0.18) or sex (F _(1,30)_ =1.14, p=0.29)). However, males responded significantly more for alcohol compared to females (main effect of sex (F _(1,30)_ =25.84, p<0.001; eta^2^=0.46) and session (F _(6,180)_ =5.97, p<0.001; eta^2^=0.17)).

The statistical analysis of the number of the earned social rewards during FR1 sessions **(Fig 5B, left)** indicated a main effect of session (two-way RM ANOVA: F _(8,232)_ =3.05, p<0.01; eta^2^=0.10) as well as the interaction ‘sex x session’ (F _(8,232)_ =2.82, p<0.01; eta^2^=0.09) but only a trend for an effect of sex (F _(1,29)_ =4.08, p=0.053; eta^2^=0.12). Similarly, for the total number of earned alcohol rewards, there was a main effect of session (F _(8,240)_ =18.08, p<0.001 eta^2^=0.38), a significant interaction between sex and session (F _(8,240)_ =3.47, p<0.001 eta^2^=0.10) but no main effect of sex (F _(1,30)_ =0.39, p=0.54). In accordance with the active lever presses analysis, males and females obtained a similar number of social reinforcers once stabilized on an FR2 schedule (**Fig 5B right**: main effect of session (F _(6,180)_ =2.82, p<0.05; eta^2^=0.09) but no effect of sex (F _(1,30)_ =1.11, p=0.30) or interaction (F _(6,180)_ =0.38, p=0.89)), but males earned almost twice more alcohol reinforcers compared to females (last 3 sessions: 35.1±1.9 and 20.5±; two-way RM ANOVA: main effect of sex (F _(1,30)_ =32.10, p<0.001, eta^2^=0.52; main effect of session: F _(6,180)_ =6.27, p<0.001, eta^2^=0.17 but no interaction: F _(6,180)_ =1.10, p=0.36).

**SUPPLEMENTARY REFERENCES**

1. Venniro, M. and Y. Shaham, *An operant social self-administration and choice model in rats.* Nat Protoc, 2020. **15**(4): p. 1542-1559.

2. Augier, E., et al., *A Method for Evaluating the Reinforcing Properties of Ethanol in Rats without Water Deprivation, Saccharin Fading or Extended Access Training.* J Vis Exp, 2017(119).

3. Augier, E., et al., *Wistar rats acquire and maintain self-administration of 20 % ethanol without water deprivation, saccharin/sucrose fading, or extended access training.* Psychopharmacology (Berl), 2014. **231**(23): p. 4561-8.

4. Lenoir, M., et al., *A choice-based screening method for compulsive drug users in rats.* Curr Protoc Neurosci, 2013. **Chapter 9**: p. Unit 9 44.

5. Augier, E., et al., *A molecular mechanism for choosing alcohol over an alternative reward.* Science, 2018. **360**(6395): p. 1321-1326.

6. Hodos, W., *Progressive ratio as a measure of reward strength.* Science, 1961. **134**(3483): p. 943-4.
